# The correlation of copy number variations with longevity in a genome-wide association study of Han Chinese

**DOI:** 10.18632/aging.101461

**Published:** 2018-06-05

**Authors:** Xin Zhao, Xiaomin Liu, Aiping Zhang, Huashuai Chen, Qing Huo, Weiyang Li, Rui Ye, Zhihua Chen, Liping Liang, Qiong A. Liu, Juan Shen, Xin Jin, Wenwen Li, Marianne Nygaard, Xiao Liu, Yong Hou, Ting Ni, Lars Bolund, William Gottschalk, Wei Tao, Jun Gu, Xiao-Li Tian, Huanming Yang, Jian Wang, Xun Xu, Michael W. Lutz, Junxia Min, Yi Zeng, Chao Nie

**Affiliations:** 1 BGI Shenzhen, Shenzhen 518083, China; 2 Center for the Study of Aging and Human Development and Geriatrics Division, Medical School of Duke University, Durham, NC 27710, USA; 3 The First Affiliated Hospital, Institute of Translational Medicine, School of Medicine, Zhejiang University, Hangzhou 310058, China; 4 Center for Healthy Aging and Development Studies, Raissun Institute for Advanced Studies, National School of Development, Peking University, Beijing 10080, China; 5 Business School of Xiangtan University, Xiangtan 411105, China; 6 The Danish Aging Research Center, Epidemiology, Biostatistics and Biodemography, Department of Public Health, University of Southern Denmark, Odense C 5000, Denmark; 7 State Key Laboratory of Genetics Engineering and MOE Key Laboratory of Contemporary Anthropology, School of Life Sciences, Fudan University, Shanghai 200433, China; 8 Department of Biomedicine, Aarhus University, Aarhus 8000, Denmark; 9 Department of Neurology, Medical Center, Duke University, Durham, NC 27704, USA; 10 School of Life Sciences, Peking University, Beijing 100080, China; 11 Department of Human Population Genetics, Human Aging Research Institute and School of Life Science, Nanchang University, Nanchang 330000, China; 12 James D. Watson Institute of Genome Sciences, Hangzhou 310058, China; 13 BGI Education Center, University of Chinese Academy of Sciences, Shenzhen 518083, China; 14 College of Medicine, University of Arkansas for Medical Sciences, Little Rock, AR 72205, USA; 15 School of Bioscience and Bioengineering, South China University of Technology, Guangzhou, China; * Equal contribution

**Keywords:** copy number variation, longevity, Han Chinese, genome association study, long-lived, middle-aged controls

## Abstract

Copy number variations (CNVs) have been shown to cause numerous diseases, however, their roles in human lifespan remain elusive. In this study, we investigate the association of CNVs with longevity by comparing the Han Chinese genomes of long-lived individuals from 90 to 117 years of age and the middle-aged from 30 to 65. Our data demonstrate that the numbers of CNVs, especially deletions, increase significantly in a direct correlation with longevity. We identify eleven CNVs that strongly associate with longevity; four of them locate in the chromosome bands, 7p11.2, 20q13.33, 19p12 and 8p23.3 and overlap partially with the CNVs identified in long-lived Danish or U.S. populations, while the other seven have not been reported previously. These CNV regions encode nineteen known genes, and some of which have been shown to affect aging-related phenotypes such as the shortening of telomere length (*ZNF208*), the risk of cancer (*FOXA1, LAMA5, ZNF716*), and vascular and immune-related diseases (*ARHGEF10, TOR2A, SH2D3C*). In addition, we found several pathways enriched in long-lived genomes, including FOXA1 and FOXA transcription factor networks involved in regulating aging or age-dependent diseases such as cancer. Thus, our study has identified longevity-associated CNV regions and their affected genes and pathways. Our results suggest that the human genome structures such as CNVs might play an important role in determining a long life in human.

## Introduction

Human lifespan has long been observed as a complex trait with approximately 25% genetic contributions [1]. To date, only very few genes have been shown consistently associated with it [2–5]. Recent studies reported that copy number variation (CNV) may directly contribute to human lifespan [6–10]. CNV is a general term for all the chromosomal rearrangements, such as deletions, duplications [11]. CNVs can change gene structures, thus affecting gene expression and phenotypes. In human, CNVs have been implicated in numerous diseases, such as autism and diabetes [12,13]. CNVs also contribute significantly to the genome instability of cancer cells [14–16]. For example, recently, Pelttari [14] reported a unique duplication encompassing most of the *RAD51* homolog C gene in breast and ovarian cancers. Habibi [15] showed that copy number changes in the gene loci, UDP Glucuronosyltransferase Family 2 members, B28 and B17 were associated with prostate cancer. In addition, Zhou and his colleagues linked 93 CNVs to hepatocellular carcinoma [16].

A few studies have investigated the association of CNV with human lifespan using genome-wide approaches [6–10]. Kuningas [7] first reported this association in 11442 human samples representing two cohorts with the ages ranging from 62.0 to 75.3 and 34.0 to 69.8. They found large deletions in 11p15.5 (*p* = 2.8×10^-6^, Hazard Ratio (HR) = 1.59) and 14q21.3 (*p* = 1.5×10^-3^, HR = 1.57) among the oldest people. Another study uncovered a deletion in the *CNTNAP4* gene in a female group of 80 years of age (Odds Ratio (OR) = 0.41, *p* = 0.007), but not the male group (OR = 0.97, *p* = 1) [9]. Recently, a study in Caucasians (n = 388; cases: 81-90 year-olds; controls: 65-75 year-olds) revealed an insertion allele of the *CNTNAP2* gene in esv11910 CNV of males (OR = 0.29, 95%; CI: 0.14–0.59; *p* = 4×10^-4^), but not females (OR = 0.82, 95%; CI: 0.42–1.57, *p* = 0.625) [10].

In this study, we investigated the association of CNVs and longevity in Han Chinese by genotyping 4007 individuals obtained from the Chinese Longitudinal Healthy Longevity Survey (CLHLS) database. We have identified a few CNVs, and most of them were new. Some of them encode the known genes and pathways that have been associated with longevity only in this study. The deletions of the cancer-causing and aging-related genes encoded in these CNVRs may extend healthy lifespan for long-lived individuals. Future research on these CNVRs will shed light on the regulatory mechanism controlling human longevity.

## RESULTS

### The numbers of CNVs increase in the genomes of long-lived individuals

To identify the CNVs associated with longevity in Chinese population, we recruited 1950 long-lived individuals called “cases”, and 2057 middle-aged Chinese as “controls. These individuals were further separated into four cohorts: 1000 long-lived and 1215 middle-aged who lived in the north of China, and the rest of 950 long-lived and 842 middle-aged who resided in the south. The two Northern cohorts were also called “discovery cohorts”, and the two Southern cohorts were referred as “replicate cohorts”.

Using the Principal Component Analysis (PCA) method, no extra sub-cluster was found between case and control comparison (Supplementary Figure 1). Our data obtained from the Northern cohorts revealed a significantly increased number of CNVs among long-lived individuals compared to the controls (6.94 ± 0.35 vs 5.96 ± 0.27, *p* = 0.027). The similar results were found in the Southern replicate cohorts (5.96 ± 0.27 vs 4.60 ± 0.17; *p* = 0.001). Total identified CNVs were summarized in detail in Table 1.

**Table 1:** Summary of CNVs.

		N	Mean age	Female%	Mean CNV length(kb)	Total CNV length(kb)	Mean Count CNV(N)	Mean DEL length(kb)	Total DEL length(kb)	Mean Count del(N)	Mean DUP length(kb)	Total DUP length(kb)	Mean Count dup(N)
Combined samples	Case	1950	101.51(90-117)	74.30	83.78±1.49	508.44±19.78	6.46±0.22	47.11±1.19	224.23±14.04	4.15±0.20	104.43±2.61	284.20±14.01	2.30±0.10
	Control	2057	48.22(30-65)	60.77	85.57±1.86	450.53±16.32	5.40±0.18	49.92±1.33	201.81±11.88	3.25±0.16	93.94±2.67	248.72±11.56	2.14±0.07
	P				0.457	**0.024**	**0.001**	0.118	0.221	**0.001**	**0.005**	0.051	0.193

**Figure 1:**
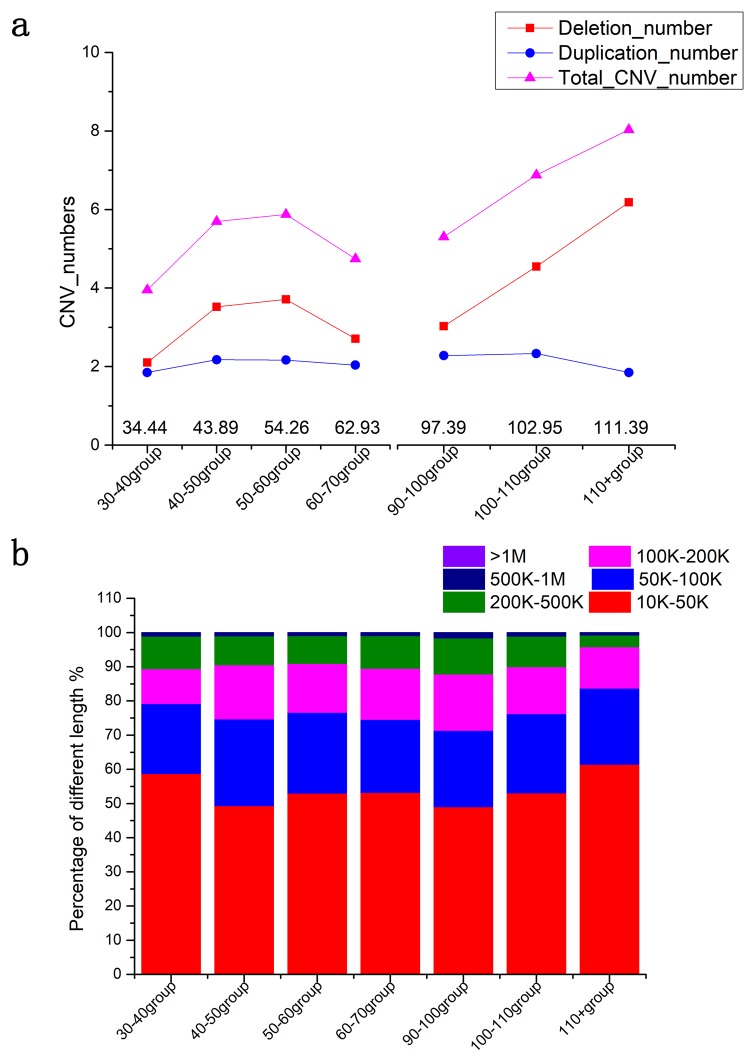
**CNV burden in different age groups.** (**a**) The numbers of CNVs in different age groups. (**b**) The added lengths of CNVs in different age groups. Triangles represent the numbers of the CNVs. The areas in each histogram represent the percentage of different lengths. * p<0.05 by t-test.

In all participating subjects including both cases and the controls, we identified 10046 deletions and 6932 duplications in total. We found that the numbers of CNVs increased significantly in older ages (Spearman rho = 0.386, *p* = 0.002; Figure 1), particularly, the deletions increased much more than the duplications (Spearman rho = 0.356, *p* = 0.004). On average, the centenarians (101.51 ± 0.07 years of age) contained 4.15 ± 0.20 deletions and 2.30 ± 0.10 duplications, showing a significant increase (*p* = 0.001) compared to the middle-aged (48.22 ± 0.16 years of age), who had 3.25 ± 0.16 deletions and 2.14 ± 0.07 duplications. The Spearman correlation was also calculated to show the significance between ages and total added lengths of CNVs (Spearman rho = 0.31, *p* = 0.017); long-lived people had 508.54 ± 19.8 kb of total CNVs, which was significantly longer than 453.69 ± 17.25 kb in middle-aged controls (*p* = 0.024). In summary, we have shown that the CNV numbers, especially the deletion numbers, increased significantly in the genomes of long-lived people.

### Identification of CNVRs associated with longevity

**Figure 2:**
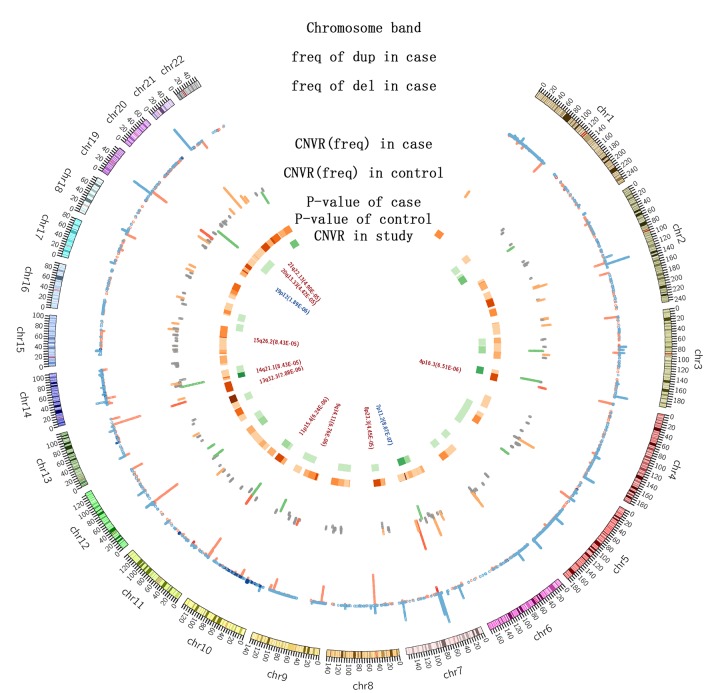
**The distribution of CNVs among 4007 individuals.** The first circle indicates the positions of chromosomal bands; the second is a histogram representing the frequencies of CNVs in long-lived individuals (red: deletions; blue: duplications; height: CNV frequencies); the third is also a histogram showing the CNV frequencies in the long-lived (orange) and middle-aged (orange) individuals. Frequency: orange and green, >1%; grey, <1%; red, the validated CNVRs by qPCR. The heatmap shows the p-values of CNVRs: orange for the long-lived, green for the middle-aged, and the color gradience towards dark indicates decreasing p-values. The text presents the names of the CNVRs identified in this study.

We identified the CNVRs associated with longevity, according to a previously published method [17]. These regions included 62 deletions and 5 duplications identified from the Northern cohorts, 21 deletions and 6 duplications in the Southern cohorts, and 99 deletions and 27 duplications in the combined samples (Figure 2). Among them, we identified eleven CNVs (2 duplications and 9 deletions) with case frequency > 1%, *p* value < 3.97×10^-4^ and length > 10k in long-lived individuals from the north as well as the north and south combined samples (Table 2, Supplementary Table 1). These CNVRs have been previously deposited in the database of genomic variants (DGV, http://dgv.tcag.ca/dgv/app/home) through other studies unrelated to longevity (Supplementary Figure 2). In order to confirm the accuracy of genotyping and CNV detecting methods, top six CNVRs (with p-value less than 10^-5^) were chose to do the quantitative PCR. (Supplementary Figure 3) and all these six CNVRs were validated accurate.

**Table 2:** The longevity CNVRs and their encoded genes.

CNVR(hg19)	Cytoband	Type	*p* value	Odds Ratio	Frequency in long-lived%	Frequency in middle-aged%	Gene encoded	CNVR Length(kb)
chr7:57787663-57839005	7p11.2	Dup	8.67×10^-7^	1.59	15.90	5.74	*ZNF716*	51.34
chr19:22145147-22231026	19p12	Dup	1.89×10^-6^	4.11	2.36	0.63	*ZNF208,ZNF257*	85.88
chr13:100620882-100630973	13q32.3	Del	2.88×10^-6^	3.88	2.41	0.63	*ZIC5,ZIC2*	10.09
chr11:2939050-2949861	11p15.4	Del	6.24×10^-6^	4.81	1.85	0.39	*PHLDA2,SLC22A18*	10.81
chr4:1610872-1621223	4p16.3	Del	6.51×10^-6^	12.21	1.18	0.10	*FAM53A*	10.35
chr9:130479233-130504070 chr20:60900481-60927421	9q34.11 20q13.33	Del Del	6.76×10^-6^ 4.42×10^-5^	21.3 10.65	1.03 1.08	0.05 0.10	*PTRH1,SH2D3C,TOR2A,TTC16* *LAMA5*	24.84 26.93
chr8:1789006-1799964	8p23.3	Del	4.45×10^-5^	10.65	1.03	0.15	*ARHGEF10*	10.96
chr21:38442208-38453183	21q22.13	Del	4.90×10^-5^	5.16	1.54	0.29	*PIGP,TTC3*	10.98
chr14:38058065-38081005	14q21.1	Del	8.43×10^-5^	4.57	1.54	0.34	*TTC6,FOXA1*	22.94
chr15:96898166-96909819	15q26.2	Del	8.43×10^-5^	4.57	1.54	0.34	*NR2F2*	11.65

### Functional analysis of eleven CNVRs

To determine if our identified CNVRs can affect biological function, we analyzed these loci for encoded genes. We found several genes within or near 11 CNVRs including Zinc Finger Protein 716 (*ZNF716)*, Zinc Finger Protein 208 (*ZNF208),* Zinc Finger Protein 257 (*ZNF257),* Zic Family Member 5 *(ZIC5),* Zic Family Member 2 *(ZIC2),* Pleckstrin Homology Like Domain Family A Member 2 *(PHLDA2),* Solute Carrier Family 22 Member 18 *(SLC22A18),* Family With Sequence Similarity 53 Member A *(FAM53A),* Peptidyl-TRNA Hydrolase 1 Homolog *(PTRH1),* SH2 Domain Containing 3C *(SH2D3C),* Torsin Family 2 Member A *(TOR2A),* Tetratricopeptide Repeat Domain 16 *(TTC16),* Laminin Subunit Alpha 5 *(LAMA5),* Rho Guanine Nucleotide Exchange Factor 10 *(ARHGEF10),* Phosphatidylinositol Glycan Anchor Biosynthesis Class P *(PIGP),* Tetratricopeptide Repeat Domain 3 *(TTC3),* Tetratricopeptide Repeat Domain 6 *(TTC6),* Forkhead Box A1 *(FOXA1) and* Nuclear Receptor Subfamily 2 Group F Member 2 *(NR2F2)* (Table 2).

Some of these genes have previously been linked to longevity. For example, it has been shown that the rs4925386 in *LAMA5* gene in 20q13.33 belongs to laminin alpha family. The age-stratified analyses showed that the rs4925386-T allele was positively associated with longevity (*p* = 0.001) [18]. The CNVR in 9q34.1 encodes genes *SH2D3C* and *TOR2A*. *SH2D3C* is a signaling adapter protein required for T cell activation, and a decrease in *SH2D3C* might cause B cell dysfunction and impaired immune function, which could affect lifespan [19]. In addition, the CNVR in 14q21.1 encodes the gene, *FOXA1*; upregulation of *FOXA1* has been shown to decrease diet-restriction-induced longevity in *C. elegans* [20]. Additionally, *NR2F2,* a gene about 14kb away from 15q26.2 (*p* = 8.43×10^-5^, OR = 4.57) showed higher expression in older samples and was related to vascular development during oxidative stress-induced cellular senescence; vascular-related disease could be used as a marker for aging people [21].

To further understand whether these CNVRs affect specific pathways regulating aging process, The Database for Annotation, Visualization and Integrated Discovery (DAVID), Gene Ontology (GO), and Functional Enrichment analysis tool (FunRich) [22–24] were used for the enriched pathway analyses (Table 3). Six pathways were found to be enriched using FunRich including FOXA1 and FOXA transcription factor networks, which were most directly related to the regulation of longevity.

**Table 3:** Longevity genes and pathways enriched in the genomes.

Database Category	Term	Genes	p-value
GOTERM_BP_FAT	epithelium development	ZIC2,ZIC5,FOXA1,LAMA5,NR2F2	1.2×10^-4^
GOTERM_BP_FAT	tube morphogenesis	ZIC2,ZIC5,FOXA1,LAMA5	4.1×10^-4^
GOTERM_BP_FAT	tube development	ZIC2,ZIC5,FOXA1,LAMA5	2.0×10^-3^
GOTERM_BP_FAT	morphogenesis of embryonic epithelium	ZIC2,ZIC5,LAMA5	2.1×10^-3^
GOTERM_BP_FAT	epithelial tube morphogenesis	ZIC2,ZIC5,LAMA5	2.8×10^-3^
Biological pathway	FOXA1 transcription factor network	FOXA1,NR2F2	1.30×10^-3^
Biological pathway	Mesenchymal-to-epithelial transition	PHLDA2,SLC22A18,FOXA1	2.16×10^-3^
Biological pathway	FOXA transcription factor networks	FOXA1,NR2F2	4.36×10^-3^
Biological pathway	Alpha6 beta4 integrin-ligand interactions	LAMA5	1.39×10^-2^
Biological pathway	Synthesis of glycosylphosphatidylinositol (GPI)	SLC22A18	2.14×10^-2^
Biological pathway	Post-translational modification: synthesis of GPI-anchored proteins	PIGP	3.26×10^-2^

To determine if the difference in CNVR could be found between the nonagenarians (90-99) and centenarians (>100), we conducted the CNVR analyses in these two groups separately We detected four new CNVRs that were unique for the nonagenarians, and six new CNVRs only in the centenarians (Supplementary Table 2).

### The variants in the CNVRs and eQTL

To determine if SNPs could alter gene expression in CNVRs, we investigated our 11 CNVRs using the Haploreg database [25]. Four of them were found to contain multiple SNPs, defining ≥10 eQTLs in tissue database that might affect gene function (Supplementary Table 4). These CNVRs are the deletions in 21q22.13, 20q13.33, and 11p15.4, and a duplication in 19p12. The eQTLs in the 21q22.13 deletion region could alter the expressions of the *PIGP* and *TTC3* genes primarily in brain tissue. According to the annotation database Genecards (http://www.genecards.org/), *PIGP* resides in the protein complex that catalyzes the transfer of N-acetylglucosamine from UDP-GlcNAc to phosphatidylinositol, the first step of the glycosylphosphatidylinositol (GPI) biosynthesis. *TTC3* is an ubiquitin-protein ligase that mediates the ubiquitination and subsequent degradation of phosphorylated AKT proteins. Several SNPs in the 20q13.33 deletion might affect the *LAMA5* and *CABLES2* gene expression. *LAMA5* has been shown previously to affect cellular adhesion, migration, and organization. Two SNPs, rs1661052 and rs450244, in the 11p15.4 deletion region, showed strong cis-eQTL characteristics and might affect the *SLC22A18* expression and its function in transporting organic cations. Finally, the SNPs located in the 19p12 duplication region can affect the expression of the ZNF gene family member such as *ZNF208* and *ZNF 257*.

### Commonly shared CNVRs between Han Chinese and other ethnicities

To determine if our identified CNVRs in Han Chinese were also found in other ethnicities, we compared our data to a Danish and an American study. The Danish study investigated 603 nonagenarians or long-lived individuals of 90.0–102.5 years of age or 96.9 on average [6], while the U.S. ‘wellderly’ healthy aging cohort included 1,354 individuals from 80 to 105 years old or 84.2 on average [26].

Among the 11 CNVRs, the duplication in 7p11.2 (Chr7: 57787663- 57839005) was interesting. The Danish study identified a similar duplication in 7p11.2 (Chr7: 57208666 - 57882950) with a population frequency of 1%. Another similar duplication was also found in the U. S. ‘wellderly’ with a population frequency of 0.2% and a 19% overlap (Table 4). To further determine the distribution of this duplication in different ethnicities, we examined the genomes in the 1000 Genomes Project, which contained randomly picked adults older than 18 years old from healthy populations of multiple ethnics. A similar duplication (Chr7: 57729553 - 57807369) was found in the phase 3 database with low frequencies or no distribution across all populations: 0.1% in European, 0.8% in African, and none in East Asian, South Asian, and mixed American (an admixed population of Americans). In contrast, the frequency of this duplication was significantly higher in long-lived individuals: 15.89%, 1%, and 0.2% in the Han Chinese, Danish, and American population, respectively. Thus, these studies strongly supported that the duplication in 7p11.2 associated with longevity.

**Table 4:** The CNVRs commonly shared in three ethnic groups.

Han Chinese						Danish					U.S. ‘wellderly’			
CNVR	Cytoband	Type	Frequency(%)	CHB	Gene encoded	CNVR	Type	Frequency(%)	EUR	gene	Overlap%	Type	Frequency(%)	AMR
Chr20:60900481-60927412	20q13.33	DEL	1.07	0	*LAMA5*	Chr20:60872280-60909518	DEL	0.33	0	20*	NA	NA	NA	NA
Chr19:22145147-22231026	19p12	DUP	2.36	0	19*	NA	NA	NA	NA		9	DUP	0.1	0

Among all the Han Chinese longevity CNVRs, two were found overlapping with the longevity CNVRs yielded from the Danish study and three from the US study (one shared in both Danish and US study). However, only one of these four attained a larger than 1% frequency. Bold indicates the CNVRs with at least 5% overlapping regions among the three long-lived ethnic groups. NA: the absence of CNVR. Frequency %: the occurrence of the CNVRs among the investigated samples. CHB, EUR, AMR represent Chinese, Europeans, and ad mix Americans in the 1000 genomes database (phase 3). 19*, 20*, and 7* indicate the regions that contain the genes, ZNF208 and ZNF257, ADRM1 and LAMA5, and GUSBP10, LOC105375297, ZNF716, and MIR3147, respectively.

Our three other CNVRs were found to be comparable with the CNVRs identified in the U.S. or Danish study (Table 4). The deletion in 20q13.33 (Chr20: 60872280 - 60909518) partially overlapped with the Danish CNVR (Chr20: 60872280 - 60909518). Its population frequencies were 1.07%, 0.33%, and 0 in Han Chinese, Danish, and the 1000 genomes database, respectively. The other two CNVRs consisted of one duplication in 19p12 (Chr19: 22145147 – 22231026) and one deletion in 8p23 (Chr8: 1789006 – 1796964). Both shared 9% and 12% overlapping regions with a similar duplication and deletion in the ‘wellderly’, respectively. The population frequencies in Han Chinese, Ad Mixed American, and the 1000 genomes database for the duplication CNVR were 2.36%, 0.1%, and 0, and 1.02%, 0.59%, and 0 for the deletion CNVR, correlatively These results also indicated that the CNV frequencies were higher in long-lived population than those represented by the 1000 Genomes Project, which composed of randomly picked adults.

## DISCUSSION

In a genome-wide association study, we investigated the genomes obtained from 1950 long-lived and 2057 middle-aged Han Chinese people and identified 11 CNVRs that were associated with longevity. Four of them had partially overlapping regions with the CNVRs uncovered from the long-lived Danish or U.S population, while the rest seven were first reported in this study. Our statistical analyses indicated that the four overlapped CNVRs in the 7p11.2, 20q13.33, 19p12, and 8p23.3 bands were the strongest candidates with p values, 8.68×10^-7^, 4.42×10^-5^, 1.89×10^-6^, and 4.45×10^-5^, respectively.

It has been well-accepted that an increasing number or total length of deletions or duplications indicate genomic instability. Forsberg et al. has shown that genome instability increases with longevity [27]. In this study, we observed a significant increase in CNV numbers between long-lived and middle aged individuals (Figure 1). The added length of total CNVs also reflected this increase. Nygaard et al. has shown that mortality had a significant increase per 10 kb of CNV length increase in long-lived individuals [6]. We hypothesize that the long-lived CNVs are not hazard, for example, the deletions of carcinogenesis-related regions may help people improve lifespan.

In this study, we identified 11 CNVRs associated with long-lived Han Chinese including 2 duplications and 9 deletions. These CNVRs might affect the expressions of 19 known genes (Table 2), some of these genes were known to regulate cellular aging process.

For example, several studies have shown that the variant rs8105767 (ZNF208), which locates 20kb away from our 19p12 CNV region, led to several diseases by shortening the length of telomeres. A population study [28] demonstrated that the mutation of this gene could cause neuroblastoma. A genome-wide meta-analysis identified seven loci affecting telomere, and one of which was the rs8105767. This variant was also shown to be involved in shortened telomeres in leucocytes and increased the risk of coronary artery disease in the European descent population [29]. Thus, these results indicated that this CNV may affect telomere, therefore, the human longevity.

*ARHGEF10* in 8p23.3 has been shown to play a key role in the RhoA signaling; an animal research demonstrated that the inactivation of *ARHGEF10* could inhibit the platelet aggregation and protect mice from thrombus formation [30]. A genome-wide association study indicated that the variant rs7862362 A>T significantly decreased the cutaneous melanoma-specific survival [31]. Interestingly, one research showed that the deletion of 8p23.3 could induce intellectual disability and delay developmental processes [32], specifically in the Chinese population. These results suggest the deletion of *ARHGEF10* may promote healthy aging by improving vascular function and suppress cancer occurrence.

The *LAMA5* gene locates in 20q13.33. An Italian GWAS study has associated its rs4925386 T allele with longevity (*p* = 0.001) and shorter stature (*p* = 0.01) [18]. *LAMA5* was reported to mediate cellular adhesion, migration, and organization [18]. Overexpression of *LAMA5* can induce colorectal cancer through KRAS, while the genetic inactivation of *LAMA5* impairs the adhesion of KRAS-mutant colorectal cancer cells [33]. It was showed in a Chinese population that the alterations of the duplication DNVR in 20q13.33 could increase the risk of ovarian endometriosis and glioma [34,35]. Thus, the deletion of this region may reduce the risk of the carcinogenesis and extend lifespan for these long-lived individuals.

Among all the 19 CNVR related genes, *FOXA1* might have the strongest effects on longevity. The *FOXA1* transcription factor network plays a key role in DNA repairing, therefore, maintaining the integrity of the genome. It has been shown that inhibiting the expression of *FOXA1* can extend the lifespan in *C.elegans* [20]. Some studies suggested that *FOXA1* was potentially an oncogene because overexpression or increased copy numbers of *FOXA1* were found in a variety of cancers [36,37]. In our samples, 1.53% of cases had deletions in *FOXA1*, which may potentially reduce the risk of cancer, leading to a healthier lifespan. It has been hypothesized that some people live longer were due to the reduced copy numbers in certain oncogene regions.

Besides their direct effects on gene structures, SNPs in CNVs can also change gene expression. We showed, in three of our identified CNVRs, that eQTLs resulting from multiple SNPs could affect the expression of nearby genes. For example, the SNPs in the 21q22.13 CNVR correlated significantly with the expression of the PIGP and TTC3 genes in brain tissue. PIGP regulates glycosylphosphatidylinositol-anchor biosynthesis in animals and has also been related to Down syndrome [38]. Variants of this gene could lead to age-dependent Alzheimer’s disease [39]. Another example is that the 20q13.33 deletion region also contains multiple SNPs that could affect the expression of LAMA5 and CABLES2 genes. A study involving a SNP from the 11p15.4 deletion region led to the discovery that SLC22A18 in this region may function as a tumor suppressor [40]. Our analyses showed that most of our identified CNVRs contain multiple SNPs, thus affecting gene expression.

In conclusion, we analyzed 4007 samples in a genome-wide association study and identified 11 CNVRs including 9 deletions and 2 duplications that were strongly associated with long-lived Han Chinese. Four of them were also found in the similar chromosomal regions in a Danish or a U.S. long-lived populations, suggesting these might be commonly shared in long-lived human. The other seven were first identified in this study. We also found that the number of deletions increased significantly with longevity. Based on our gene and pathway analysis results, we conclude that some of our identified CNVRs encode cancer-causing or aging-related genes, and the deletions of these regions may extend healthy lifespan for long-lived individuals. Future research on these CNVRs will shed light on the regulatory mechanisms controlling human longevity.

## SIGNIFICANCE

In this study, we analyzed copy number variations in a longevity cohort of Han Chinese. A total of 4,007 samples, obtained from 1950 long-lived and 2057 middle-aged healthy individuals, were investigated. By comparing these two age-groups, we identified 126 CNV regions (CNVRs) that significantly increased in the long-lived populations. Eleven among these 126 CNVRs were enriched significantly among long-lived individuals with multiple test p-values less than 3.97×10^-4^ and population frequency larger than 1%. Strikingly, four among these eleven CNVRs were found to partially overlap with the CNVRs identified in American ‘Wellderly’ and Danish longevity cohort studies. Annotation of these CNVRs revealed nineteen genes in these regions; some of them have been previously shown to function in the pathways regulating aging process. Our findings suggested that the genomic structure changes such as CNVs might contribute to the complex traits of longevity by altering gene expression.

## METHODS

### The population studied

Our study included 1950 long-lived cases and 2057 middle-age controls from the Chinese Longitudinal Healthy Longevity Survey (CLHLS), which represented 22 provinces of China, following the method described previously [5,41; http://web5.pku.edu.cn/ageing/html/datadownload.html).

These people were divided, based on their geographical locations, into two population cohorts, representing the discovery and replicate samples. The former was composed of 1000 long-lived cases and 1215 middle-age controls from 11 provinces in the north of China, while the latter consisted of 950 long-lived and 842 middle-aged individuals from 11 provinces in the south of China.

### The whole genome genotyping

Genotyping was carried out following the standard protocols of the Illumina HumanOmniZhongHua-8 BeadChips (Illumina Inc.). A beadchip contains 900,015 SNPs markers with a mean and median spacing of 3.3 kb and1.7kb, respectively. Quality control (QC) was conducted using plink 1.06 and call rate > 0.95; 339 samples from the replicate was excluded. Principal component analysis (PCA) was also subsequently performed using plink [42].

### CNV detection

CNVs were detected using the software PennCNV 1.04 [43]. In the software, a hidden Markov model (HMM) determined the CNVs with the total signal intensity Log R ratio (LRR) and B allele frequency (BAF) for each SNP marker. The LRR and BAF values can be downloaded from the Illumina Genomestudio2011. The population frequency of the B allele (PFB) was calculated using the BAF values collected from the discovery or replicate study. The GC file was obtained from UCSC genome bioinformatics (http://hgdownload.cse.ucsc.edu/goldenPath/hg19/database/gc5Base.txt.gz). The final CNVs were obtained after excluding those that met these conditions: LRR SD (standard deviation) > 0.3, GC wave factor > 0.05, CNV number >100, CNV length < 10kb, consecutive SNPs < 10, and confidence <10.

### CNVs encoded gene annotation and pathway analysis

The centenarian specific CNVs were annotated using human genome reference version 19 (hg19) within the range of 100kb. Genome ontology (GO) enrichment was performed using Go Ontology (http://geneontology.org/) [23]. Functional enrichment and interaction network analysis of genes and proteins were performed using The Database for Annotation, Visualization and Integrated Discovery (DAVID) (https://david.ncifcrf.gov/home.jsp) [24] and Functional Enrichment analysis tool (FunRich) 3.1.3 [25].

### Determination of the CNVRs associated with longevity

We compared the long-lived to the middle-aged individuals to identify CNVs associated with longevity using the CNVR searching software, ParseCNV20 [17]. The CNVs were called based on p values calculated by Fisher’s exact test. The SNPs in the CNVRs were also compared among the old and middle-aged groups with Fisher’s test.

The identified CNVRs were then annotated based on the hg19 reference genome. Those CNVs overlapping with telomeres were discarded.

We then compared our identified CNVs with a Danish study (personal communications with Dr. Nygaard) and a U.S. study (personal communications with Dr. Ali Torkamani).

### The validations of CNVRs

Quantitative polymerase chain reaction (qPCR) was used to validate CNVRs. Considering the long lived DNA are valuable, limited samples could be used for the validation. In addition, we still need samples for proceeding the next stage of GWAS experiments, so the top six CNVRs (with p-value less than 10^-5^) were chose to do the quantitative PCR and confirm the accuracy of genotyping and CNV detecting methods. Primers sets were designed for each of selected CNVRs (Supplementary Table 3). The qPCR conditions followed the ABI Step One (Life technologies Inc.) in 96-well plates: 95°C for 10 min to denature DNA samples, followed by 40 cycles (95°C for 15 s and then 58°C for 1 min). Each sample was repeated three times. The relative copy number (RCN) was calculated using CT values (2^–∆∆Ct^). RCN was then adjusted by setting the no-copy-number-change as “1”. Larger than “1.2” was regarded as duplication, while smaller than “0.8” indicated deletion.

### Expression Quantitative Trait Loci (eQTL) analysis

Haploreg V4.1, a web-based tool for annotating noncoding genomic regions for variations was used to examine the effects of SNPs on gene expression and determine eQTLs (http://archive.broadinstitute.org/mammals/haploreg/haploreg.php). These eQTLs from Haploreg were also identified from the GTEx Project (https://www.gtexportal.org/home/) and Geuvadis Data Browser (https://www.ebi.ac.uk/Tools/geuvadis-das/).

### Statistical analysis of CNV burden

To determine if the CNV numbers or total added lengths of CNVs were statistically different between the long-lived and middle-aged cohorts, a Bonferroni correction independent students’ t test was applied. The Spearman correlation was calculated between CNV numbers and CNV lengths. Since genetic association studies are often confronted by the problem of complex population structure that can result in false positive results, in this study, we used an adjusted p-value of 3.97×10^-4^ (0.05/126), based on multiple testing, as a cutoff to identify statistically significant in long-lived specific CNVRs. The t test analyses were performed using SPSS Statistics 19.0 software (SPSS, Inc.) and p<0.05 was regarded as significant. Data correlation analyses were performed using a R software from the R Project for Statistical Computing (3.1.4). Figure 1 and supplement figures were constructed with Origin 8.5, a graphing software (Origin Lab Inc.), and Figure 2 was by Circos 0.69, a software package for visualizing genomic data and information in circular layout (http://circos.ca/).

## Supplementary Material

Supplementary File

Supplementary Table 4
