# Supplementary material for: The correlation of copy number variations with longevity in a genome-wide association study of Han Chinese
Source: Aging (Albany NY). 2018 Jun 5;10(6):1206–22. doi: 10.18632/aging.101461 (PMC6046244; doi:10.18632/aging.101461)
Supplement: Supplementary File [file aging-10-101461-s001.pdf]

SUPPLEMENTARY MATERIAL

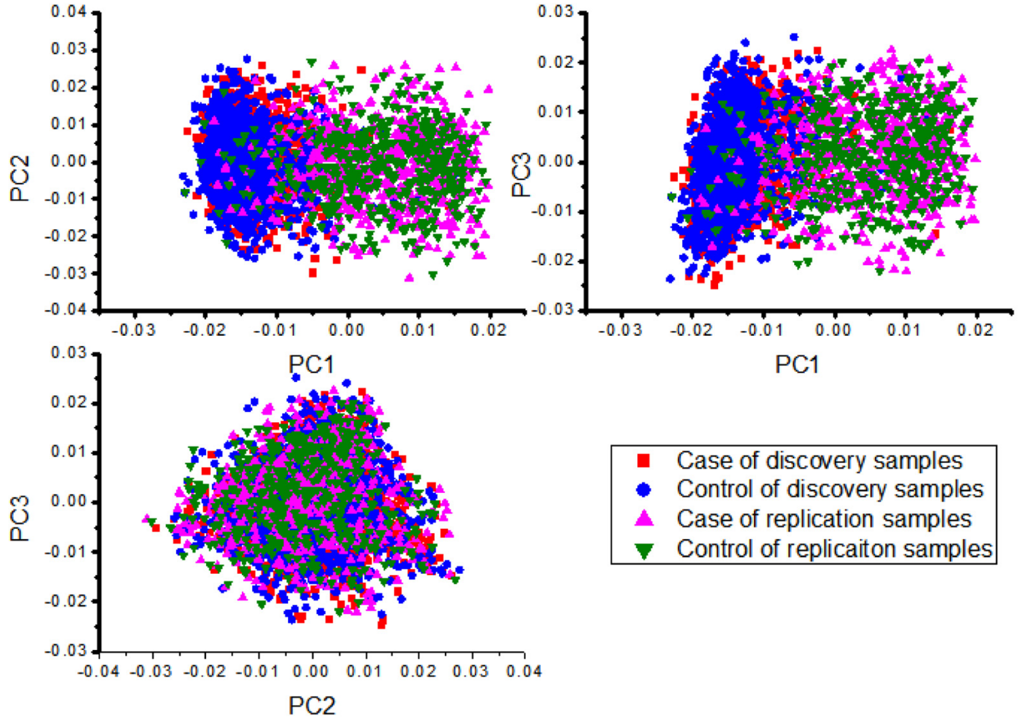

Supplementary Figure 1. Principal component analyses of the discovery and replicate cohorts: different symbol represented different groups: circle: case of discovery samples; square: control of discovery samples; up-triangle: case of replication samples; down-triangle: control of replication samples.

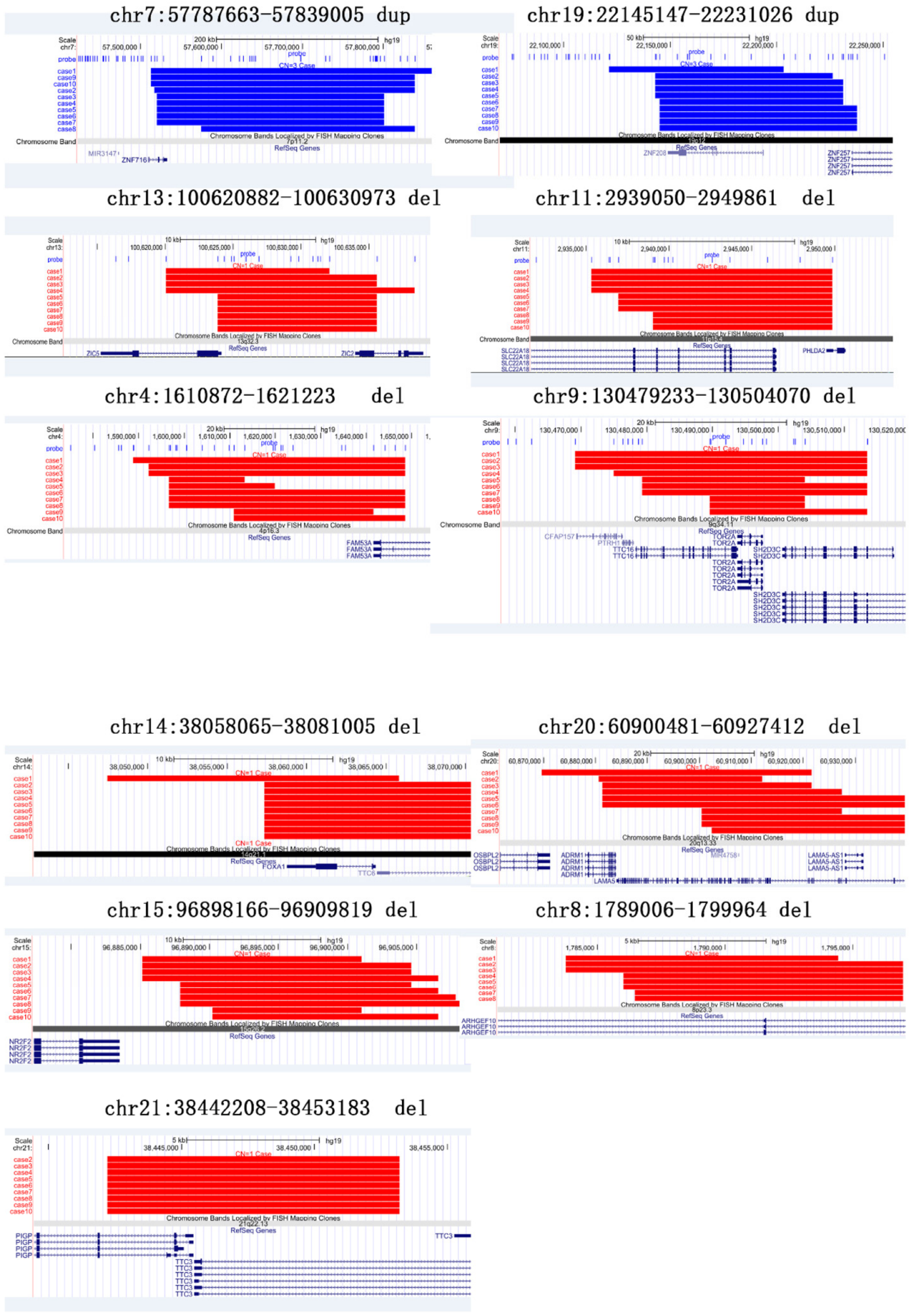

Supplementary Figure 2. The chromosomal display of the longevity-associated CNVRs. Red rectangles represent deletions in cases while blue represent duplications.

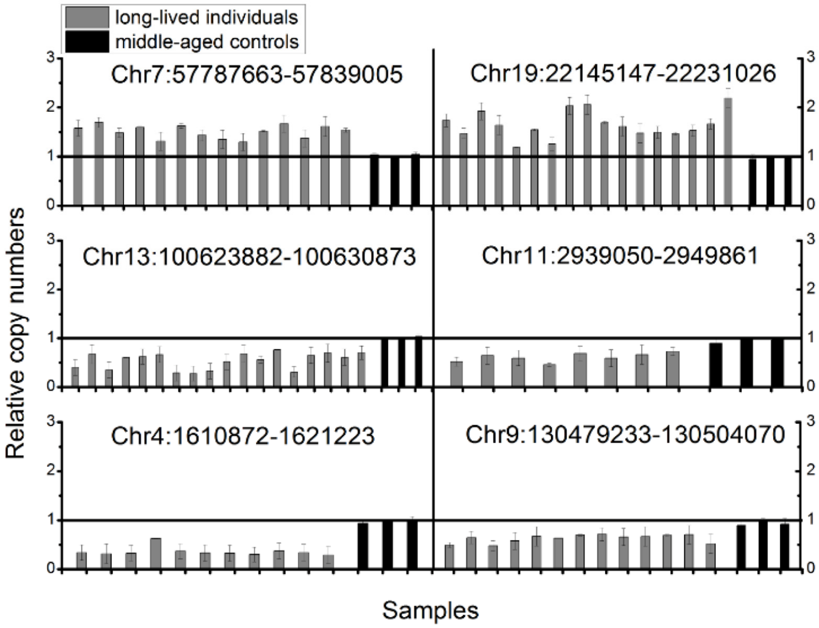

Supplementary Figure 3. qPCR validation of CNVR. Two independent primers were designed and used for the validation; better results were shown in Supplement Figure 3. Relative copy number (CN) was calculated with the CT value of each sample. The relative copy number for the control was 1. More or less than 1 was regarded as the duplications or deletions.

Supplementary Table 1. Deletions and duplications commonly identified in the discovery, discovery replicate, and their combined cohorts.

	Discovery samples					Replication samples				Combined samples			
CNVR	Type	P	OR	Case%	Control%	P	OR	Case%	Control %	P	OR	Case%	Control %
chr9:130479233-130504070	DEL	6.11×10^{-6}	22.25	1.80	0.08	NA	NA	NA	NA	NA*	NA*	NA*	NA*
chr4:1610872-1621223	DEL	1.30×10^{-5}	20.99	1.70	0.08	NA	NA	NA	NA	NA*	NA*	NA*	NA*
chr7:57787663-57839005	DUP	5.28×10^{-5}	1.69	16.0	5.6	1.77×10^{-3}	1.68	11.79	7.36	8.68×10^{-7}	1.59	15.89	5.74
chr20:60900481-60927412	DEL	5.73×10^{-5}	18.49	1.60	0.08	NA	NA	NA	NA	NA*	NA*	NA*	NA*
chr11:2939050-2949861	DEL	1.25×10^{-4}	4.61	2.60	0.58	1.30×10^{-2}	8.95	1.05	0.12	6.25×10^{-6}	4.81	1.84	0.39
chr15:96898166-96909819	DEL	4.2×10^{-4}	5.86	1.90	0.33	NA	NA	NA	NA	NA*	NA*	NA*	NA*
chr8:1789006-1799964	DEL	1.24×10^{-3}	7.99	1.30	0.25	1.66×10^{-2}	infinity	1.04	0	4.45×10^{-5}	10.65	1.02	0.15
chr19:22145147-22231026	DUP	2.07×10^{-3}	3.9	1.90	0.58	4.13×10^{-4}	4.71	2.74	0.59	1.89×10^{-6}	4.11	2.35	0.63
chr13:100620882-100630973	DEL	2.94×10^{-3}	2.96	2.40	0.82	2.06×10^{-4}	6.94	2.42	0.36	2.89×10^{-6}	3.88	2.41	0.63
chr21:38442208-38453183	DEL	3.95×10^{-3}	4.19	1.80	0.41	4.01×10^{-3}	10.76	1.26	0.12	4.90×10^{-5}	5.16	1.53	0.29
chr14:38058065-38081005	DEL	1.29×10^{2}	2.98	1.70	0.58	3.03×10^{-4}	infinity	1.37	0	8.43×10^{-5}	4.57	1.53	0.34

Copy number variant region (CNVR), The discovery samples CNV position with information about chromosome and start and stop base pair positions based on the GRCh37 hg19 genome build; DEL, deletion; DUP, duplication; Case%, case frequency; Control%, control frequency; OR, odds ratio; P, P-value obtained from a fisher's exact test for longevity and middle aged subjects. NA, not applicable due to a frequency lower than 1% in the replication study; NA*, not applicable due to a frequency lower than 1% in the replication samples, but get a frequency higher than 1% in the combined samples.

Supplementary Table 2. Association between specific deletions and duplications among nonagenarians, centenarians and middle-aged control.

CNVR(hg19)	P	case%	control%	Gene	Length(kb)	CNVtype
**90-99 case enrich vs 100+ control**						
CNVR(hg19)	P	case%	control%	Gene	Length(kb)	CNVtype
chr16:60082486-60100445	9.74E-03	12.64	8.66	LOC644649	17.96	Deletion
chr19:22142930-22237629	1.21E-03	2.83	0.77	ZNF208,ZNF257	94.70	Duplication
chr11:89319607-89397819	1.57E-03	1.89	0.35	AB231784,FOLH1B,NOX4	78.21	Duplication
chr13:102315051-102326456	3.84E-02	3.02	1.48	ITGBL1	11.41	Duplication
**100+ case enrich vs 90-99 control**						
CNVR(hg19)	P	case%	control%	Gene	Length(kb)	CNVtype
chr14:22755575-22767645	5.93E-05	4.08	0.75	AV4S1,T-Cell Receptor V-alpha region,TCR-alpha,TCRA,TRA,TRA@,TRAC,TRD	12.07	Deletion
chr9:130489558-130513469	3.67E-03	1.41	0.00	SH2D3C,TOR2A,TTC16	23.91	Deletion
chr21:46936423-46952750	5.68E-03	1.20	0.00	SLC19A1	16.33	Deletion
chr20:60881330-60909832	2.20E-02	1.41	0.19	ADRM1,LAMA5,MIR4758	28.50	Deletion
chr16:1858888-2074219	3.49E-02	1.27	0.19	C16orf73,FAHD1,GFER,HAGH,HS3ST6,NDUFB10,NOXO1,NPW,RNF151,RPL3L,RPS2,SEPX1,SNHG9,SYNGR3,TBL3,ZNF598	215.33	Deletion
chr2:25835636-25886631	4.21E-02	1.13	0.00	DTNB	51.00	Deletion
**<65 case enrich vs 100-120 control**						
CNVR(hg19)	P	case%	control%	Gene	length(kb)	CNVtype
chr11:2939050-2949861	6.25E-06	1.75	0.56	PHLDA2,SLC22A18	10.81	Deletion
chr4:1610872-1621223	6.51E-06	1.12	0.14	FAM53A	10.35	Deletion
chr20:60900481-60927412	4.42E-05	1.02	0.14	LAMA5,MIR4758	26.93	Deletion
chr21:38442208-38453183	4.90E-05	1.46	0.42	PIGP,TTC3	10.98	Deletion
chr14:38058065-38081005	8.43E-05	1.46	0.49	C14orf25,FOXA1	22.94	Deletion
chr1:1151232-1191870	1.99E-04	1.02	0.21	B3GALT6,FAM132A,SDF4,UBE2J2	40.64	Deletion
chr16:88786245-88806348	2.27E-04	1.07	0.35	AK294743,PIEZO1	20.10	Deletion
chr17:80367353-80391684	2.40E-04	1.17	0.35	C17orf101,HEXDC	24.33	Deletion
chr2:176985696-177011655	2.40E-04	1.17	0.35	AX747372,BC047605,HOXD8,HOXD9,LOC100506783	25.96	Deletion
chr6:158634175-158655732	6.53E-03	1.46	0.99	GTF2H5	21.56	Deletion
chr2:239047020-239064760	1.76E-02	1.07	0.63	KLHL30	17.74	Deletion
chr7:57787663-57839005	8.68E-07	15.07	15.35	ZNF716	51.34	Duplication
chr19:22145147-22231026	1.89E-06	2.24	0.92	ZNF208	85.88	Duplication
chr4:190801108-190894359	1.03E-02	2.77	2.46	BC087857,FRG1	93.25	Duplication
**100-120 case enrich vs <65 control**						
CNVR(hg19)	P	case%	control%	Gene	length(kb)	CNVtype
chr1:1151232-1191870	4.47E-05	1.34	0.15	B3GALT6,FAM132A,SDF4,UBE2J2	40.64	Deletion
chr11:2939050-2949861	3.79E-07	2.32	0.39	PHLDA2,SLC22A18	10.81	Deletion
chr12:132923159-132956191	1.50E-05	1.20	0.10	GALNT9	33.03	Deletion
chr14:38057334-38081005	5.47E-05	1.69	0.34	C14orf25,FOXA1	23.67	Deletion
chr16:2065666-2079393	8.81E-05	1.27	0.15	NPW,SLC9A3R2,TCRBV20S1	13.73	Deletion
chr2:128387937-128403997	3.31E-05	1.13	0.10	GPR17,LIMS2,MYO7B	16.06	Deletion
chr2:176992220-177011655	3.74E-04	1.27	0.24	AX747372,BC047605,HOXD8,LOC100506783	19.44	Deletion
chr2:239047020-239066379	5.92E-03	1.48	0.53	KLHL30	19.36	Deletion
chr20:60900481-60927412	2.97E-06	1.41	0.10	LAMA5,MIR4758	26.93	Deletion
chr21:38442208-38453183	4.23E-06	1.90	0.29	PIGP,TTC3	10.98	Deletion
chr3:103079255-103096303	3.26E-03	1.20	0.34	DD413615	17.05	Deletion
chr6:158634175-158647999	1.47E-02	1.48	0.73	GTF2H5	13.82	Deletion

chr7:27229549-27264337	3.49E-04	1.06	0.15	HOTTIP,HOXA13	34.79	Deletion
chr9:130479233-130504070	1.97E-07	1.41	0.05	PTRH1,SH2D3C,TOR2A,TTC16	24.84	Deletion
chr9:139980385-140028291	8.81E-05	1.27	0.15	DPP7,LOC100289341,MAN1B1	47.91	Deletion
chr19:22145147-22231026	1.92E-03	1.69	0.63	ZNF208	85.88	Duplication
**<65 case enrich vs 90-99 control**						
CNVR(hg19)	P	case%	control%	Gene	length(kb)	CNVtype
chr1:238930549-238987220	4.96E-03	1.22	0.00	LOC339535	56.67	Duplication
chr10:42406669-42467126	3.24E-02	2.09	0.94	LOC441666	60.46	Duplication
90-99 case enrich vs <65 control						
CNVR(hg19)	P	case%	control%	Gene	length(kb)	CNVtype
chr13:100624374-100635606	4.48E-03	2.08	0.63	ZIC2	11.23	Deletion
chr9:7017391-7036325	7.16E-03	1.13	0.19	KDM4C	18.93	Deletion
chr2:176985696-176999012	1.31E-02	1.13	0.24	AX747372,HOXD8,HOXD9	13.32	Deletion
chr6:158634175-158655732	1.84E-02	1.89	0.68	GTF2H5	21.56	Deletion
chr13:84120973-84157927	3.62E-02	1.70	0.68	SLITRK1	36.95	Deletion
chr5:46128475-46160697	3.68E-02	1.89	1.31	HCN1	32.22	Deletion
chr14:44507227-44533909	4.49E-02	2.08	0.97	FSCB	26.68	Deletion
chr19:22145147-22237629	2.29E-08	4.15	0.63	ZNF208,ZNF257	92.48	Duplication
chr11:89319607-89397819	5.52E-05	1.89	0.24	AB231784,FOLH1B,NOX4	78.21	Duplication

Supplementary Table 3. The primer sequences used for qPCRs.

CNVR	Primer-F	Primer-R
chr7:57787663-57839005	AGGACAAAACACTTCTGAGCC	TGACCAAAAACATACAGCCAA
chr19:22145147-22231026	GCCGAAAGCAGTTTTTACCA	TTTGTCTGTGCATGTCTTTCC
chr13:100620882-100630973	TTTGGAAACGACGAGTCCTT	TTCAGAGAGGAAACGATGACA
chr11:2939050-2949861	TCTGAGTGTCACCACTGCCTT	TCCGTGGCTCGCTATCCTAT
chr4:1610872-1621223	AAGAAGACAGGCAGCTCCTCA	TCTGCTGCTGGCACATGGA
chr9:130479233-130504070	TTCAGATCCTTCTGTAGGGGA	TCTCTGGAAGACCACATCCTT

Please browse the Full Text version to see the data of **Supplementary Table 4. eQTL functional annotation.**
